# Detection of low frequency variants of the *NLRP3* gene in “mutation- negative” CAPS patients using massive parallel sequencing

**DOI:** 10.1186/1546-0096-13-S1-P34

**Published:** 2015-09-28

**Authors:** J Thiem, M Lesche, A Dahl, A Kränkel, J Roesler, A Rösen-Wolff

**Affiliations:** 1 University Hospital Carl Gustav Carus, Department of Pediatrics, Dresden, Germany; 2 BIOTEC TU Dresden, Deep Sequencing Group SFB655, Dresden, Germany

## Introduction

Recent studies showed a notable frequency of somatic mosaicism of the *NLRP3* gene in chronic, infantile, neurological, cutaneous and articular (CINCA) syndrome (35%) and Muckle-Wells syndrome (MWS)(12.5%) patients that were not detectable by common Sanger sequencing.

## Objectives

We are currently trying to detect and quantify low frequency *NLRP3* variants in mutation-negative patients, who suffer from a CINCA Syndrome, MWS or Familial Cold Autoinflammatory Syndrome (FCAS) or show cryopyrin-associated periodic syndrome (CAPS)-like symptoms without a classical phenotype.

## Methods

The exons of the *NLRP3* gene were amplified via PCR from genomic PBMC DNA. The obtained PCR products were sequenced with an Illumina HiSeq platform. For SNP calling we used the GATK pipeline of the 1000 Genome Project, if the coverage attained 40,000 fold. In order to prove the accuracy of the method, we quantified dilutions of a known heterozygous mutation (T348M) mixed with wildtype DNA. For the correlation between the test results and the phenotype of the patients we developed a survey including symptoms and medical treatment.

## Results

In one CINCA patient we detected a new *NLRP3* variant (L359S) in 30% of the sequence. Using a cut-off of 5% mutated DNA sequences, we did not detect any other mutation of the *NLRP3* gene in the other 47 samples we tested so far. We tried to increase the sensitivity by establishing a new statistic method (M+2SD), setting the cut-off at 0.5%. This led to a drastic reduction of specificity with irreproducible results.

## Conclusion

Massive parallel sequencing is a reliable method for the quantification of low frequency variants of the *NLRP3* gene. Increasing the sensitivity (<5% cut-off) results in detecting PCR artifacts and a dramatic loss of specificity. The probability of somatic mosaicism in mutation-negative CAPS patients is higher if the symptoms are more severe. Although 35% of mutation-negative CINCA patients and 12.5% of mutation-negative MWS patients harbor somatic mutations, this seems to be extremely rare in patients with CAPS-like symptoms without classical CAPS phenotype.

## Acknowledgements

This study was supported by Novartis Pharma GmbH, Nürnberg (ARW); BMBF Projekt NGSgoesHPC (ML) and SFB655 (DFG) (AD, AK).

